# Lifetime Carcinogenic Risk Proportions from Inhalation Exposures in Industrial and Non-Industrial Regions

**DOI:** 10.3390/ijerph182413295

**Published:** 2021-12-16

**Authors:** Vítězslav Jiřík, Ladislav Tomášek, Ivana Fojtíková, Tomáš Janoš, Markéta Stanovská, Pavlína Guňková, Andrea Dalecká, Adéla Vrtková, Radim J. Šrám

**Affiliations:** 1 Centre for Epidemiological Research, Faculty of Medicine, University of Ostrava, Syllabova 19, 703 00 Ostrava, Czech Republic; janos.tomas@gmail.com (T.J.); stanovska.marketa@seznam.cz (M.S.); pavlina.gunkova@osu.cz (P.G.); dalecka.andrea@gmail.com (A.D.); adela.vrtkova@vsb.cz (A.V.); radim.sram@osu.cz (R.J.Š.); 2 Department of Epidemiology and Public Health, Faculty of Medicine, University of Ostrava, Syllabova 19, 703 00 Ostrava, Czech Republic; 3 National Radiation Protection Institute (SURO), Bartoškova 28, 140 00 Prague, Czech Republic; ladislav.tomasek@suro.cz (L.T.); ivana.fojtikova@suro.cz (I.F.); 4 Department of Applied Mathematics, Faculty of Electrical Engineering and Computer Science, VSB-Technical University of Ostrava, 708 00 Ostrava, Czech Republic

**Keywords:** radon, benzo[a]pyrene, lung cancer, occupational exposure, lifetime cancer risk

## Abstract

The aim of this work was to estimate the share of selected significant risk factors for respiratory cancer in the overall incidence of this disease and their comparison in two environmentally different burdened regions. A combination of a longitudinal cross-sectional population study with a US EPA health risk assessment methodology was used. The result of this procedure is the expression of lifelong carcinogenic risks and their contribution in the overall incidence of the disease. Compared to exposures to benzo[a]pyrene in the air and fibrogenic dust in the working air, several orders of magnitude higher share of the total incidence of respiratory cancer was found in radon exposures, for women 60% in the industrial area, respectively 100% in the non-industrial area, for men 24%, respectively 15%. The share of risks in workers exposed to fibrogenic dust was found to be 0.35% in the industrial area. For benzo[a]pyrene, the share of risks was below 1% and the share of other risk factors was in the monitored areas was up to 85%. The most significant share in the development of respiratory cancer in both monitored areas is represented by radon for women and other risk factors for men.

## 1. Introduction

Cancer of respiratory tract (trachea, bronchus, and lung cancer–further stated LBC) is the world’s most common cause of death among all cancer diseases [1]. In the Czech Republic, lung cancer is the third most common cause of death of all diseases and the most common cause of death of all cancer diseases [2]. The occurrence of LBC is influenced by many risk factors including outdoor air pollution, indoor air pollution, occupational exposure, genetic predisposition and many of lifestyle risk factors, especially tobacco smoking [3].

Outdoor and indoor air pollution concentrations differ significantly across different parts of the world and also across the Czech Republic (CR) [4]. Although the respirable fraction of particulate matter PM 2.5, which are emitted from various natural and anthropogenic sources is most associated with premature mortality of LBC [5,6], the polycyclic aromatic hydrocarbons, which are linked to PM 2.5, and other genotoxic substances increased carcinogenic potential of inhaled air pollution, where the significant part is probably caused by the proven human carcinogen benzo[a]pyrene [7], whose inhalation exposure is associated with LBC and also with upper respiratory tract cancer and esophageal cancer [8].

The Czech Republic is also one of the European countries with the highest concentrations of indoor radon. Radon is found in all buildings in various quantities and the specific concentration in the house is related to the amount of radon present in the subsoil under the building [9]. Radon is a proven human carcinogen that irradiates the epithelium of respiratory system through the inhalation exposure and probably is the second most important cause of lung cancer after smoking. The effect of radon on the incidence of lung cancer has been convincingly demonstrated by epidemiological studies. Studies have also shown that the combined effects of radon and smoking increase the harmful effects on health [9,10].

Professions at risk of development of LBC include mining workers, quarrying professions, professional asbestos exposure or coking plant workers. In the Czech Republic there is a register of occupational diseases which has been a reliable source of databases of this information since 1991 [11].

Tobacco smoking is the most significant modifiable risk factor from lifestyle risk factors, the main consequence of smoking is the development of respiratory diseases (especially LBC) and cardiovascular diseases, including other diseases. The incidence of cancer, including LBC, and the premature mortality associated with tobacco smoking are considered a global problem [12]. There are also other risk factors (like genetic predispositions, infectious diseases, other lifestyle risk factors, socioeconomic influences) that cause development of LBC [3].

The aim of the study was to quantify lifetime cancer risks of LBC (see International Classification of Diseases ICD10–C33, C34) from selected risk factors related to inhalation exposures and their quantitative comparison.

Another aim of the study was to compare the individual contributions of the risk factors between environmentally differently polluted regions.

**Figure 1:**
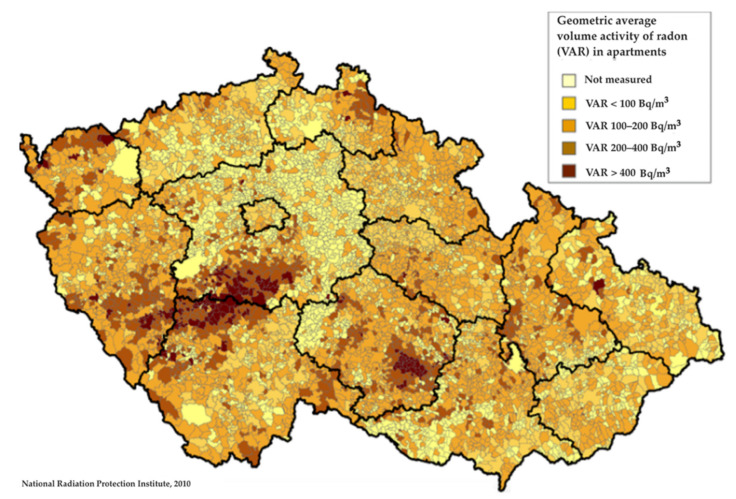
Geometric average volume activity of radon in apartments [9].

**Figure 2:**
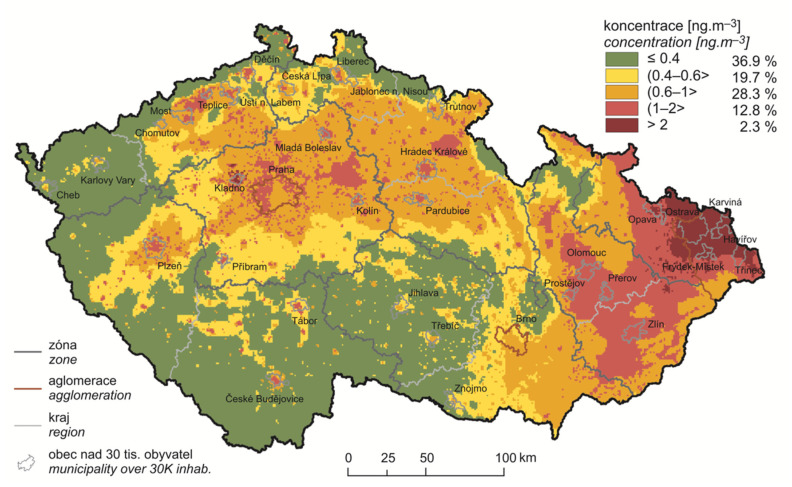
Five-year average of annual average concentrations of benzo[a]pyrene in 2014–2018 [13].

The volume activity of radon in apartments in the Czech Republic is shown in Figure 1. Below Figure 2 shows the five-year average annual average concentrations of benzo[a]pyrene in the period 2014–2018.

## 2. Materials and Methods

The general procedure of methodology for achieving the objectives of the work was the estimation of lifetime cancer risk according to two methods, those of the United States Environmental Protection Agency (US EPA) and the International Agency for Research on Cancer (IARC)/International Commission on Radiological Protection (ICRP). The procedure was based on processing all available historical data on population exposure in two environmentally differently polluted regions and their comparison with all available data on the occurrence of LBC in these regions of interest. 

### 2.1. Input Data

#### 2.1.1. Study Population

The study population was selected from two areas of the Czech Republic (CR) in the monitored period 1994–2016. The first (four districts of Moravian-Silesian region of CR, two districts–Bruntál and Frýdek Místek were excluded due to the mountainous nature of the landscape and not completely fitting into an industrial or environmentally polluted region, which in the following text is marked IA–industrial area) has been considered for a long time as one of the most polluted areas in Central and Western Europe and also the most polluted area in the CR in terms of air quality. There is a population of 942,873 inhabitants (with most inhabitants in Ostrava: 338,548). The second area (all seven districts of South-Bohemian region of CR, which in the following text is marked NA–non-industrial area) with a population of 626,419 inhabitants, the most in České Budějovice with 182,977) is considered one of the least environmentally polluted areas of CR [4]. Population numbers are the annual arithmetic average of the population (1996–2016) in the monitored regions. The Czech Republic has a total population of 10.65 million and the above numbers of inhabitants in the regions of interest are given according to the Czech Statistical Office [14].

#### 2.1.2. Incidence Rates

Data on new numbers of LBC cases (diagnoses C33 and C34, i.e., lung, bronchus and trachea cancer according to ICD10) were obtained from a nationally guaranteed database of diseases in the Czech Republic, which is managed by the Institute of Health Information and Statistics [15]. Firstly, for each year in the range 1994–2016, the crude incidence rate was calculated as the proportion of the absolute number of cases and population for whole population and with respect to gender. Then, the mean of the crude incidence rates over the years 1994–2016 was calculated for whole population and with respect to gender (CIR, see Table 1).

For each year in the range 1994–2016, the age-specific incidence rates (i.e., crude incidence rates in every age group 0–4, 5–9, 10–14, 15–19, 20–24, 25–29, 30–34, 35–39, 40–44, 45–49, 50–54, 55–59, 60–64, 65–69, 70–74, 75–79, 80–84, 85+) were calculated. Then, the 2019 Czech population was considered as the standard population and the age-specific incidence rates were used for calculation of the age-standardized incidence rate for each year from the range 1994–2016. The annual mean of the calculated age-standardized incidence rates from 1994–2016 was used for the description and comparison of regions and districts (SIR, see Table 1).

Lastly, for each year (1994–2016), the age-specific incidence rates per 1 inhabitant of the standard population in each age group (0–4, 5–9, 10–14, 15–19, 20–24, 25–29, 30–34, 35–39, 40–44, 45–49, 50–54, 55–59, 60–64, 65–69, 70–74) were calculated. The mean of these age-specific incidence rates through the years was taken and the cumulative incidence rate, i.e., the sum of the means multiplied by 5 (the age-specific incidence rates were computed for 5-year age intervals) is taken as representation of the total lifetime risk of LBC for a 75-year-old (LR_o_, see Table 1).

**Figure 3:**
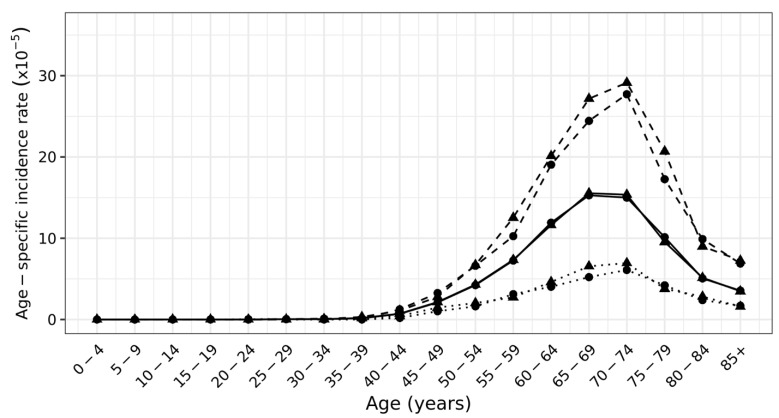
Annual mean gender-specific age-specific standardized incidence rates per 1 inhabitant, standardization using 2019 Czech population. Solid—total population, dashed—male, dotted—female, circle—South Bohemia region, triangle—Moravian–Silesian region.

The occurrence of newly diagnosed cases of the LBC disease depending on the age group is visualized in Figure 3. The onset of the LBC disease after 40 years of age with maximum in the age group 70–74 years indicates the duration of lifetime exposures.

#### 2.1.3. Risk Factors and Exposure Assessment

The four types of exposures were considered, respectively four risk factors to which the population (or part of the population) is exposed, and which may cause LBC.

##### Radon

The first of the considered risk factors was indoor inhalation exposure to carcinogenic radon in apartments and buildings. These exposures were represented by the detected volume activities of radioactive radon and by the mean ambient concentration of benzo[a]pyrene in airborne dust. The radiation load of radon was determined by the method of integral trace dosimetry with passive detectors, within the Radon Program under the auspices of the National Radiation Protection Institute in cooperation with the State Office for Nuclear Safety in the Czech Republic. In this work, it is assumed that the value of radon volume activity was approximately constant throughout the observed period 1994–2016, i.e., currently ideally for a 75-year-old man since 1945. This assumption is based on the fact that the radon concentration in the monitored regions can be considered relatively constant [9]. Although this monitoring was performed only during one whole year, it can therefore be assumed that it represents several decades.

##### Benzo[a]pyrene

The second risk factor was represented by modeled concentrations of benzo[a]pyrene in the air. Time series data of air pollution were used for model calculations, which were taken from a valid national database of average territorial concentrations published by the Czech Hydrometeorological Institute [16]. The average annual concentrations were interpreted from the available tabular overviews of data from air pollution monitoring stations and modeled area 5-year average concentrations in a regular network of squares (1 × 1 km). By combination of these data based on mutual relations of individual air pollution characteristics, the average annual concentrations in the entire regular network of squares were interpreted. The missing data were replaced by a linear trend determined by the closest known values. This is how the data for the period 1997–2019 were processed. Subsequently, a less accurate estimate of these concentrations was made for the years 1980, 1985, 1990, 1995 based on emission balance data for individual evaluated districts. Based on the statistical analysis, the resulting data were finally corrected for the inhabited areas of the districts of the monitored regions. Since it was necessary to obtain an approximation of air pollution concentrations for all years from the interval, based on the data obtained by the procedure described above, the approximation was performed using a linear trend based on the closest known values and back extrapolation until 1945 was performed using a constant trend (see Table 2).

##### Fibrogenic Dust

The third risk factor was occupational exposure to fibrogenic dust containing silica in a large group of miners and other professions in IA (in the Moravian Silesian region) [17]. Data on the incidence of pneumoconiosis recognized as an occupational disease in these professions were obtained from electronic yearbooks of occupational diseases which were taken over from the National Institute of Public Health in the Czech Republic for the period 1996–2016 [11]. Other source data include data on the proportion of cancer in occupational pneumoconiosis from a Czech cohort study and the proportion of LBC from cancer in employees with pneumoconiosis [18].

##### Others Risk Factors

The fourth and also last risk factors are all other possible causes of LBC, e.g., genetic predisposition, infectious diseases, other lifestyle factors, socioeconomic influences that cause lung cancer [3]. These other possible causes of LBC have not been studied in this work.

### 2.2. Data Processed

#### 2.2.1. Lifetime Exposure

The method for expressing the lifetime cumulative effective dose of radiation from radon and its daughter products per 1 inhabitant is the following (Equation (1)):

(1) LD=VAR·ED·CF·OF·RCF

where:

LD—lifetime cumulative effective dose (mSv) per 1 inhabitant;
VAR—constant volume activity of radon in the observe period (Bq/m^3^);
ED—exposure duration (75 years);
CF—conversion factor (365.25 × 24 h);
OF—occupancy factor (0.7);
RCF—radon dose conversion factor (6.70 × 10^−6^ mSv/Bq·hours·m^−3^).

In the air pollution, an insignificant radon load was assumed and the average person’s stay in the indoor environment was considered to be 70% of the day; see occupancy factor (OF) [19]. The radon dose conversion factor is derived from epidemiological studies [20]. 

The method for expressing the lifetime average exposure concentration from long-term concentration of benzo[a]pyrene in ambient air is the following (Equation (2)):

(2) $$\mathrm{LC}=\frac{c_{\mathrm{avg}}\cdot \mathrm{ED}}{\mathrm{AT}}$$

where:

LC—lifetime average exposure concentration (µg/m^3^);
c_avg_—average air pollution concentration (µg/m^3^) replaced by an approximation for all years and the approximation using a linear trend based on the closest known and back extrapolation (1945–2019);
ED—exposure duration (75 years);
AT—average lifetime (80 years).

#### 2.2.2. Lifetime Risk

Overall lifetime risk of LBC from all risk factors (all causes) is represented by the cumulative incidence rate (LR_o_, see Table 1). This lifetime risk LR_o_ can be divided into the risks of individual risk factors x. In our case, given that a total of five risk factors were considered (see above), the LR_o_ can be written (Equation (3)):

(3) $${\mathrm{LR}}_{o}={\mathrm{LR}}_{1}\cdot {\mathrm{LR}}_{2}\cdot {\mathrm{LR}}_{3}\cdot {\mathrm{LR}}_{4}$$

where:

LR_o_—lifetime risk of LBC from all risk factors for a 75-year-old (i.e., the cumulative incidence rate);
LR_1_—lifetime risk of LBC from exposure to radon;
LR_2_—lifetime risk of LBC from exposures to benzo[a]pyrene;
LR_3_—lifetime risk of LBC from occupational exposure;
LR_4_—lifetime risk of LBC from others risk factors.

The lifetime risk of LBC for radon exposure was estimated by using the relationship between exposures and the likelihood of cancer occurrence. For radon we derive for exposure assessment according to US EPA [21] and at the same time from ICRP [22]. Thus, two approaches can be distinguished in part in the assessment of lifetime risk [23]. For radon, the lifetime risk in the indoor environment of apartments and buildings was calculated as follows (Equation (4)):

(4) $${\mathrm{LR}}_{1}=\mathrm{LD}\cdot {\mathrm{UCR}}_{\mathrm{Rn}}$$

where:

LD—lifetime cumulative effective dose of radon (mSv);
UCR_Rn_—unit cancer risk for radon 5.11 × 10^−5^ (Sv)^−1^ according to US EPA [21];
UCR_Rn_—unit cancer risk for radon 4.75 × 10^−5^ (Sv)^−1^ according to ICRP [22].

For benzo[a]pyrene and other air pollutants the unit cancer risks are published by the World Health Organization (WHO) [24] and by the United States Environmental Protection Agency (US EPA) [25]. Results are expressed in lifetime cancer risk (LCR). Unit cancer risk (UCR) used to calculate lifetime cancer risk represent the increase in risk per unit exposure or dose and describe the relationship between the likelihood of new cancer occurring depending on the lifetime dose [26]. Significant differences in the estimation of air pollution risks using the US EPA and WHO approaches are due to the two-order difference in UCR for benzo[a]pyrene. To express benzo[a]pyrene exposure we use the lifetime average exposure concentration which for this calculation also includes the so-called pulmonary clearance (back expiration, metabolism, excretion etc.) and no cumulative effective dose as in the case of radon (see above). In radon, pulmonary clearance does not occur due to the short half-life of radon. Dose from radon and his progeny is delivered to the lung tissues before clearance can take place, that occurs absorption into blood or by particle transport to the alimentary tract [27]. The method for expressing the lifetime cancer risk for benzo[a]pyrene is the following (Equation (5)):

(5) $${\mathrm{LR}}_{2}=\mathrm{LC}\cdot {\mathrm{UCR}}_{\mathrm{BaP}}$$

where:

LC—lifetime average exposure concentration of benzo[a]pyrene (ng/m^3^);
UCR_BaP_—unit cancer risk for benzo[a]pyrene 6.00 × 10^−4^ (µg/m^3^)^−1^ according to US EPA [8];
UCR_BaP_—unit cancer risk for benzo[a]pyrene 8.7 × 10^−2^ (µg/m^3^)^−1^ according to WHO [28].

To calculate the lifetime risk of LBC (LR_3_) in employees of the above professions, firstly the average annual number of oncological diseases (including LBC) in employees with pneumoconiosis in the monitored regions (ANC) was calculated from average annual number of pneumoconiosis recognized as an occupational disease in 1996–2016 (ANP) [11], and the average proportion of cancer (in addition to LBC also cancer of colon, kidney, stomach and bladder) in employees with pneumoconiosis obtained from Czech cohort study (PCP) for the period 1996–2016 [18] (Equation (6)).

(6) ANC=ANP·PCP

Furthermore, it was possible to use ANC and other data from Czech cohort study [18]—the average proportion of lung cancer from all cancers in employees with pneumoconiosis (PLC)—to estimate the average annual number of LBC in employees/miners (ANL) with pneumoconiosis in the areas of interest (Equation (6)):

(7) ANL=ANC·PLC

The proportion of the average annual number of LBC in employees/miners with pneumoconiosis (ANL) and the average population (POP) in the observed period in the years 1996–2016 expresses the average annual risk of LBC (ARL) which can be attributed to occupational (employee) exposures in the monitored regions (expressed to the whole population) (Equation (8)).

(8) $$\mathrm{ARL}=\frac{\mathrm{ANL}}{\mathrm{POP}}$$

It is possible to express the lifetime risk of LBC (LR_3_) (for a 75-year-old person) by multiplying ARL by its age (Equation (9)):

(9) $${\mathrm{LR}}_{3}=\mathrm{ARL}\cdot 75$$

where:

ANP—the average annual number of pneumoconiosis (1996–2016) in monitored regions [11];
PCP—the average proportion of all cancers in employees with pneumoconiosis [18];
ANC—the average annual number of all cancers in employees with pneumoconiosis;
PLC—the average proportion of LBC from all cancers in employees with pneumoconiosis [18];
ANL—the average annual number of LBC in employees with pneumoconiosis;
POP—the average population in monitored regions in years 1996–2016;
ARL—the average annual risk of LBC from occupational exposures (expressed for whole population in the region);
LR_3_—lifetime risk of LBC from occupational exposure (expressed on whole population in the region) for 75 old persons.

The lifetime risk of lifestyle factors and others impacts (see Section 2.1) is estimated as a difference between the total (entire) lifetime risk (LR_o_) and sum of lifetime risks from exposures to radon, benzo[a]pyrene and occupational exposure (Equation (10)).

(10) $${\mathrm{LR}}_{4}={\mathrm{LR}}_{0}-\left({\mathrm{LR}}_{1}+{\mathrm{LR}}_{2}+{\mathrm{LR}}_{3}\right)$$

#### 2.2.3. Lifetime Risk Proportion

The attributable fraction for the population (PAF) is the proportion of cases in the population that are attributable to the risk factor. In general, it is the proportion of the disease incidence in the whole study population (SIR see above) reduced by the disease incidence in the unexposed population to the risk factor and the disease incidence in the whole population. The expression of PAF for risk factor “x” using lifetime risk can then be written (Equation (11)):

(11) $${\mathrm{PAF}}_{x}=\frac{{\mathrm{LR}}_{o}-{\mathrm{LR}}_{\mathrm{neexp}\;\mathrm{to}\;x}}{{\mathrm{LR}}_{o}}$$

A part of the incidence of LBC, i.e., the lifetime risk of LBC for the unexposed population (to any risk factor considered in our study) cannot be determined from the available data. Therefore, PAF cannot be simply expressed. However it is possible to determine the proportion of lifetime risk of individual risk factor, “x” to total lifetime risk of LBC (LR_o_), referred to here as lifetime risk proportion (LRP_x_) (Equation (12)):

(12) $${\mathrm{LRP}}_{x}=\frac{{\mathrm{LR}}_{x}}{{\mathrm{LR}}_{o}}$$

where x is the designation of the risk factor (1 to 4). At this point it should be noted that LR_x_ is not equal to (LR_o_ − LR_neexp to x_), therefore LRP_x_ is not equal to PAF_x_.

## 3. Results

Volume activities of radon and benzo[a]pyrene air pollution concentrations are in Table 2, lifetime risks in Table 3. Values in these tables are no gender-specific, because exposures and risks are the same for men and women. *p*-value of the Mann—Whitney test indicates a statistically significant difference between the observed regions (IA–industrial region, NA–non-industrial region) for benzo[a]pyrene and radon exposure.

**Figure 4:**
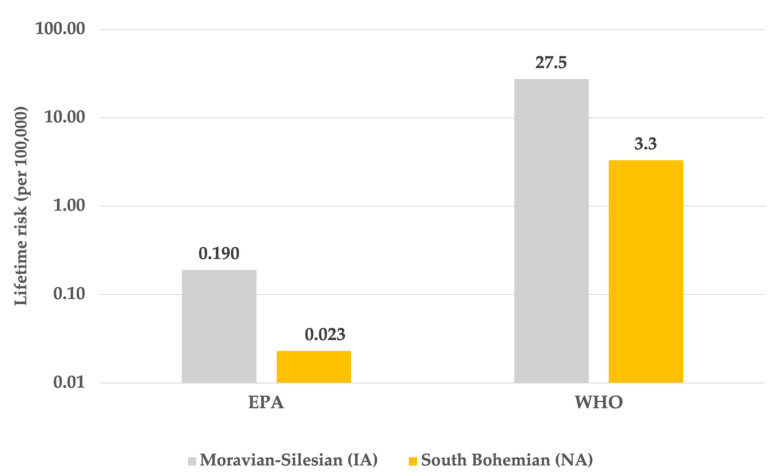
Difference between lifetime risk for benzo[a]pyrene (LR_2_) by the United States Environmental Protection Agency (EPA) and World Health Organization (WHO).

Figure 4 shows the differences between the carcinogenic risks predicted by the US EPA and IARC (WHO).

Table 4 shows the lifetime carcinogenic risk proportions for radon and benzo[a]pyrene. 

Table 5 shows the input data, the calculated lifetime risks and risk proportions for occupational exposures. 

**Figure 5:**
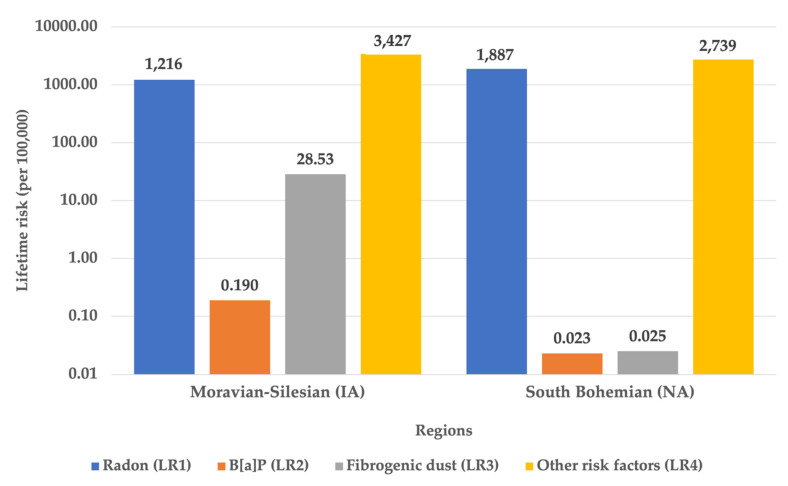
Overview of lifetime cancer risks.

A graphical overview of the lifetime carcinogenic risks of observed risk factors is given in Figure 5. The data given here for radon and benzo[a]pyrene are listed according to EPA (see above).

**Figure 6:**
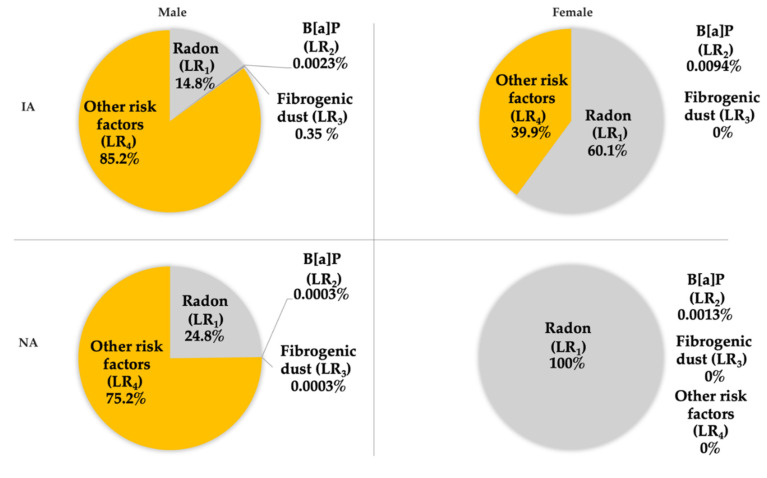
Lifetime risk proportions (gender-specific).

Figure 6 summarizes the results of lifetime cancer risks of all evaluated risk factors that were achieved. The data given here for radon and benzo[a]pyrene are according to the EPA (see above). For radon and benzo[a]pyrene, the risk proportions (shares) of the total LBC incidence are calculated using the conversion factor, respectively the EPA carcinogenic risk units.

## 4. Discussion

In this study, two regions were selected based on the objectives of the project Healthy Aging in the Industrial Region. The first region is the Moravian–Silesian Region, an industrial area (IA). There is relatively polluted air and other social structures of the population due to the types of employment, including expected different lifestyles. The second region is a non-industrial area (NA), which is the South Bohemian region, which in this sense was considered an industrially unpolluted area.

Carcinogenic risk assessment was performed in a population that showed a relatively uniform distribution by gender, slightly exceeding the number of women (see Section 2.1). The onset of LBC disease in the study population can be observed from approximately 45 years of age (see Figure 3) with the highest values in the age category 70–74 years, which agrees with the reported median age of first diagnosis of 70 years [3]. The incidence of LBC per 100,000 inhabitants in the industrial area (IA) and the non-industrial area (NA) is significantly higher in men (8190 in IA, 7595 in NA) than in women (2022 in IA, 1747 in NA). No statistically significant differences in the incidences thus expressed between areas IA and NA were found (see Table 1).

Although the incidence of LBC in IA and NA did not differ significantly, lifetime exposures of selected risk factors were different. Radon exposure was approximately 1.5 time higher in NA (77 Bq/m^3^ in IA, 120 Bq/m^3^ in NA), while benzo[a]pyrene exposure was approximately eight times higher in IA (3.378 ng/m^3^ in IA, 0.406 ng/m^3^ in NA) (see Table 2). There was also a significant difference in occupational exposures in employees who are exposed to fibrogenic dust at their workplaces. In IA, the share of these exposures is dominant, while in NA, these professions are almost non-existent. These exposures lead to pneumoconiosis in some of these workers and to LBC as an occupational disease in some workers (see Table 5). 

The population-weighted average volume activity of radon in the NA moved averaged around 120 Bq/m^3^, with the highest values in the districts of Strakonice (204 Bq/m^3^), the lowest in the district of Tábor (120 Bq/m^3^). In IA, the concentration of radon in the dwellings was significantly lower, around 77 Bq/m^3^. Comparing the above exposures with data in the literature, it can be stated that the exposure to radon in the monitored areas is relatively high compared to data from other European (or other) countries. For example, J. D. Appleton records in his publication from 2007 an average radon exposure value of 20 Bq/m^3^ in the UK, 46 Bq/m^3^ in the US and 108 Bq/m^3^ in Sweden [29]. From these radon exposure data, lifetime carcinogenic risks (LR_1_) in the IA area were estimated to be around 1200 × 10^−5^ (i.e., 1200 newly expected cancers per 100,000 inhabitants during an individual’s life of 75 years), whereas in the NA the carcinogenic risk was significantly higher, around 1800 × 10^−5^ (see Table 3).

Long-term (lifetime) exposures benzo[a]pyrene, especially in industrial area, can also be considered as one of the highest in Europe (average long-term concentration 3.4 ng/m^3^ with a maximum of 8.2 ng/m^3^ in Ostrava, see Table 2), in comparison with other states or regions [30]. The higher occurrence of radon in NA is due to the different geological subsoil in Bohemia (NA) compared to the Silesian region (IA). On the contrary, long-term concentrations of benzo[a]pyrene are naturally significantly higher in the industrial area of Silesia compared to the relatively environmentally unburdened area (average long-term concentration 0.4 ng/m^3^, see Table 2). After processing these exposure data, it was possible to state that lifelong respiration of benzo[a]pyrene represents significantly higher risks (LR_2_) in IA compared to NA with different values using carcinogenic risk units by EPA and WHO IARC (see Table 3 and Figure 4). The average value of lifetime risk according to EPA was 0.19 × 10^−5^, while according to WHO IARC it was 27.5 × 10^−5^, which is more than a two-order difference. The WHO IARC still advocates the design of a carcinogenic risk directive by a major study in the workplace after exposure of workers to coke oven emissions [28], while the EPA evaluates far more evidence and it may be more plausible to estimate this risk [8]. The results of the B[a]P exposure estimation are based only on the measured BaP concentrations, and it can be reasonably assumed that the carcinogenic potential of all air pollutants is higher in the real situation, due to the content of other cariogenic PAHs or other substances.

The data obtained of exposures to fibrogenic dust are completely dependent on the character of the studied area and depend on employment in many professions where such exposures occur. In our case, the share of exposures in the industrial area was almost 100%. The construction of risks (LR_3_) was based on the state register of occupational diseases and previously performed studies in risk professions (see Section 2.2), where it was possible to evaluate the lifetime risks of LBC from the data obtained in this way. In IA, this risk was 28.5 × 10^−5^ for the population (see Table 5), which can already be considered a significant risk as it exceeds the generally acceptable risk level of 10^−6^ [31].

It is a widely accepted fact that smoking is one of the most important risk factors for lung cancer [32]. Despite all efforts, the authors failed to obtain relevant data on smoking in individual areas of the Czech Republic, only reliable data on smoking in the population in the Czech Republic as a whole were obtained. The lifetime carcinogenic risks of respiratory cancer could not be assessed, and smoking was included in the so-called other risk factors, together with genetic predispositions, infectious diseases of the respiratory system, nutrition, physical activity, other lifestyle factors and psycho-socio-economic influences or determinants. health (see Section 2.2) [3]. The value of this ““other”” lifetime carcinogenic risk (LR_4_) ranged from 2739 per 100,000 inhabitants in the non-industrial area to 3426 per 100,000 inhabitants in the industrial area, which are certainly significant values.

The aim of the work was mainly to show the share and comparison of individual contributions to the total lifetime risk or incidence of LBC. The shares (proportions) of lifetime risks of LBCs (LRP_1_, LRP_2_, LRP_3_ and LRP_4_) and the actual incidence of this disease can be seen in Table 4 and Table 5 and Figure 6. The highest (100%) proportion was found for radon (LRP_1_) in women in NA. It is evident that this share is probably overestimated because the share of other risk factors cannot be zero. Although smoking was not evaluated in our work for the above reasons, it is known that women in the Czech Republic smoke slightly less than men, but the share of women smokers in the total number of women is about 20.7% [33], i.e., exposure to tobacco smoke would naturally have to be reflected in the occurrence of LBC. The share of the risk of radon exposure in the occurrence of LBC in men in both areas (in IA 15%, in NA 25%) is, therefore, probably lower than shown in Figure 6 in this work according to ICRP (see Section 2.2).

The contribution of the lifetime carcinogenic risk of benzo[a]pyrene to the occurrence of respiratory system (LRP_2_) is also questionable due to an inconsistent view of its carcinogenic potential, see above. However, one of the other most important ““findings”” of this work is the fact that the carcinogenicity of polluted open air is most likely to be significantly lower (even taking into account the content of other carcinogenic substances) than in the indoor environment of apartments with radioactive radon. The highest estimated share (proportion) of the lifetime carcinogenic risk of benzo[a]pyrene in the incidence of LBC was 0.6% in IA by using the carcinogenic risk directive two orders of magnitude higher of WHO IARC rather than the directive by EPA (see Table 4).

The contribution of carcinogenic risks of fibrogenic dust in the work environment (LRP_3_) to the total incidence of LBC appears to be consistent, as it has been determined from real data. For IA with high employment in high-risk occupations, it was 0.35% and may, therefore, be significant in other industrial regions (see Table 5). The contribution of other risk factors (LRP_4_) to the incidence of respiratory cancer was found to range from 0% for women in NA (see radon risk overestimation discussed above) to 85% for men in IA (see Figure 6). Given these values, it is evident that the share of smoking and other lifestyle factors plays one of the primary roles in respiratory cancer and is certainly higher than in the share of air pollution. On the other hand, it should be recalled that the estimation of this share is likely to be burdened by high uncertainty for the reasons set out above.

## 5. Conclusions

By assessing the lifetime carcinogenic risks of certain risk factors (radon in apartments, benzo[a]pyrene in the open air and fibrogenic dust in the work environment) of lung, bronchial and tracheal (LBC) cancer, the proportions of these risks and the actual incidence of this disease could be assessed. The absolute (100%) share of radon risk in the total incidence of LBC in women living in environmentally friendly areas (with clean air) seems to be overestimated, because there are credible data on a significant share of women smokers in the Czech population, which should be reflected on the occurrence of LBC. For this reason, the identified shares of radon risks in the incidence of this disease are probably lower than shown in the work. The share of carcinogenic risks of benzo[a]pyrene in the incidence of LBC is also loaded with great uncertainty due to two-orders differences in published EPA and WHO IARC carcinogenic risk guidelines, but this share is significantly lower than for radon. If the population has high employment in high-risk occupations associated with exposure to fibrogenic dust, the contribution of these risks to the overall incidence of LBC may be significant. Some recommended corrective measures to reduce the accumulation of radon in apartments, reduce the impact of lifestyle risk factors, including smoking, and improve air quality, are already being implemented. This study draws attention to large differences in the share of these risks and thus possible changes in corrective action and prevention priorities, respectively, in order to set targets to reduce risk. Specific corrective actions were not the aim of this study.

## Figures and Tables

**Table 1:** Incidence of trachea, bronchus, and lung cancer (LBC).

Region and Districts	CIR ^a^			SIR ^b^			LRo ^c^		
	Total	Male	Female	Total	Male	Female	Total	Male	Female
Moravian-Silesian (IA)	61.1	94.6	31.4	75.4	136.9	33.1	4671.62	8189.49	2021.74
Karviná	64.9	103.0	34.1	80.4	151.3	34.6	5035.17	9058.79	2191.98
Nový Jičín	51.3	79.5	22.3	67.0	118.0	24.4	4045.14	6973.28	1528.64
Opava	56.3	89.2	22.7	70.2	129.2	24.4	4356.91	7750.49	1524.20
Ostrava	64.9	97.7	38.5	77.8	137.7	40.3	4814.85	8255.79	2358.01
South Bohemian (NA)	63.0	93.3	28.1	75.5	126.8	29.6	4626.42	7595.26	1746.47
České Budějovice	60.9	79.3	28.5	74.8	112.5	30.1	4570.86	6489.86	1803.11
Český Krumlov	61.3	88.7	33.6	84.0	139.2	38.9	5261.90	8563.57	2445.38
Jindřichův Hradec	65.4	104.2	21.9	77.7	145.2	23.7	4723.50	8229.25	1485.94
Písek	71.1	111.2	35.2	78.6	137.8	34.4	4719.71	8642.65	1875.69
Prachatice	58.4	92.6	23.3	73.9	129.4	26.8	4687.91	8498.61	1654.19
Strakonice	56.3	87.0	25.6	64.9	110.5	26.3	4140.63	6901.24	1491.72
Tábor	66.9	103.4	28.9	75.8	131.0	29.4	4562.21	7876.52	1618.21
Difference ^d^	−1.9	1.3	3.3	−0.1	10.1	3.5	45.21	594.22	275.26
*p*-value ^e^				0.927	0.927	0.925			

^a^ CIR—the mean of crude incidence rates per 100,000 inhabitants from years 1994–2016. ^b^ SIR—the mean of the age-standardized incidence rates per 100,000 inhabitants from years 1994–2016, standardization using the 2019 Czech population. ^c^ LR_o_—lifetime risk of LBC from all risk factors for a 75-year-old (i.e., the cumulative incidence rate). ^d^ Difference between areas IA and NA. ^e^ *p*-value of the Mann–Whitney test of difference between areas IA and NA.

**Table 2:** Lifetime exposure to radon and benzo[a]pyrene.

Region and Districts	Radon		Benzo[a]pyrene	
	Radon Activity (Bq/m^3^) ^a^	LD (mSv) ^b^	C_avg_ (ng/m^3^) ^c^	LC (ng/m^3^) ^d^
Moravian-Silesian (IA)	77	238	3.378	3.167
Karviná	72	222	2.989	2.802
Nový Jičín	92	284	1.142	1.070
Opava	88	271	1.163	1.090
Ostrava	69	213	8.217	7.704
South Bohemian (NA)	120	369	0.406	0.381
České Budějovice	93	287	0.440	0.412
Český Krumlov	94	290	0.357	0.335
Jindřichův Hradec	125	385	0.440	0.412
Písek	160	493	0.440	0.412
Prachatice	128	395	0.395	0.370
Strakonice	204	629	0.334	0.313
Tábor	89	274	0.437	0.410
Difference ^e^	−43	−131	2.972	2.786
*p*-value ^f^	0.012		0.010	

^a^ Population-weighted mean of the districts in the region. ^b^ Lifetime cumulative dose for a 75-years-old. ^c^ The mean ambient concentration (1945–2019). ^d^ Lifetime exposure concentration for a 75-years-old. ^e^ Difference between industrial area (IA) and non-industrial area (NA). ^f^ *p*-value of the Mann-Whitney test of difference between areas (IA and NA).

**Table 3:** Lifetime risk for radon and benzo[a]pyrene non-gender specific.

Region and Districts	Lifetime Risk (per 100,000)					
	Radon		Benzo[a]pyrene		Radon + B[a]P	
	LR_1_^a^		LR_2_^b^		LR_1_ + LR_2_^c^	
	EPA	ICRP	EPA	WHO	EPA	WHO
Moravian-Silesian (IA)	1216	1130	0.190	27.55	1216	1158
Karviná	1134	1055	0.168	24.38	1135	1079
Nový Jičín	1450	1347	0.064	9.312	1450	1357
Opava	1387	1289	0.065	9.484	1387	1298
Ostrava	1087	1011	0.462	67.02	1088	1078
South Bohemian (NA)	1887	1754	0.023	3.313	1887	1758
České Budějovice	1465	1362	0.025	3.588	1465	1366
Český Krumlov	1481	1377	0.020	2.916	1481	1380
Jindřichův Hradec	1970	1831	0.025	3.588	1970	1834
Písek	2521	2343	0.025	3.588	2521	2347
Prachatice	2017	1875	0.022	3.220	2017	1878
Strakonice	3214	2988	0.019	2.724	3214	2991
Tábor	1402	1304	0.025	3.568	1402	1307
Difference ^d^	−671.3	−624.0	0.167	24.24	−671.1	−599.8
*p*-value ^e^	0.012		0.010		0.012	

^a^ Lifetime risk of LBC from exposure to radon. ^b^ Lifetime rick of LBC from exposure to benzo[a]pyrene. ^c^ Lifetime risk of LBC from exposures to radon and benzo[a]pyrene. ^d^ Difference between areas (IA and NA). ^e^ *p*-value of the Mann–Whitney test of difference between areas (IA and NA).

**Table 4:** Lifetime risk proportion non-gender specific.

Region and Districts	LRP_1_ and LRP_2_ (%)					
	EPA			WHO		
	Rn	B(a)P	Rn + B[a]P	Rn	B[a]P	Rn + B[a]P
	LRP_1_^a^	LRP_2_^b^	LRP1 + LRP_2_^c^	LRP_1_^a^	LRP_2_^b^	LRP_1_ + LRP_2_^c^
Moravian-Silesian	26.03	0.004	26.03	24.20	0.590	24.79
Karviná	22.53	0.003	22.53	20.94	0.484	21.43
Nový Jičín	35.84	0.002	35.84	33.31	0.230	33.54
Opava	31.82	0.002	31.83	29.58	0.218	29.80
Ostrava	22.58	0.010	22.59	20.99	1.392	22.38
South Bohemian	40.79	0.000	40.79	37.92	0.072	37.99
České Budějovice	32.06	0.001	32.06	29.80	0.079	29.88
Český Krumlov	28.15	0.000	28.15	26.16	0.055	26.22
Jindřichův Hradec	41.70	0.001	41.70	38.76	0.076	38.84
Písek	53.41	0.001	53.42	49.65	0.076	49.73
Prachatice	43.02	0.000	43.02	39.99	0.069	40.06
Strakonice	77.63	0.000	77.63	72.16	0.066	72.23
Tábor	30.74	0.001	30.74	28.57	0.078	28.65
Difference ^d^	−14.76	0.004	−14.76	−13.72	0.518	−13.21
*p*-value ^e^	0.109	0.006	0.109	0.109	0.006	0.109

^a^ Lifetime risk proportion (LRP_1_) from exposure to radon. ^b^ Lifetime risk proportion (LRP_2_) from exposure to benzo[a]pyrene. ^c^ Lifetime risk proportion (LRP) from exposure to radon and benzoapyrene. ^d^ Difference between areas (IA and NA). ^e^ *p*-value of the Mann–Whitney test of difference between areas (IA and NA).

**Table 5:** Occupational exposures, lifetime risks and risk proportions.

	Occupational Exposure	
	Male	
	Moravian Silesian (IA)	South Bohemian (NA)
POP—Annual arithmetic mean of the population (1996–2016) in monitored regions	942,873	626,419
ANP—The average annual number of pneumoconiosis (1996–2016) in monitored regions	85.4	0.05
PCP—The average proportion of all cancers in employees with pneumoconiosis	14%	14%
ANC—The average annual number of all cancers in employees with pneumoconiosis	11.96	0.01
PLC—The average proportion of LBC cancer of all cancer in pneumoconiosis	30%	30%
ANL—The average annual number of LBC in employees with pneumoconiosis	3.59	0.0021
ARL—The average risk of LBC from occupational exposures (per 100.000)	0.34	0.00034
LR_3_—Lifetime risk of LBC from occupational exposure (per 100.000)	28.53	0.025
LRP_3_—Lifetime risk proportion from occupational exposure	0.35%	0.00033%

## Data Availability

Data are available from the corresponding author upon reasonable request.

## Abbreviations

ANC	The average annual number of all cancers in employees with pneumoconiosis
ANL	The average annual number of LBC in employees with pneumoconiosis
ANP	The average annual number of pneumoconiosis (1996–2016) in monitored regions
ARL	The average annual risk of LBC from occupational exposures (expressed for whole population in the region)
AT	Average lifetime
B[a]P	Benzo[a]pyrene
c_avg_	Average air pollution concentration
CF	Conversion factor
CIR	Crude incidence rate
CR	Czech Republic
ED	Exposure duration
IA	Industrial area
IARC	International Agency for Research on Cancer
ICRP	International Commission on Radiological Protection
LBC	Cancer of respiratory tract (trachea, bronchus, and lung cancer)
LC	Lifetime average exposure concentration
LC	Lifetime average exposure concentration of benzo[a]pyrene
LCR	Lifetime cancer risk
LD	Lifetime cumulative effective dose of radon
LR_1_	Lifetime risk of LBC from exposure to radon
LR_2_	Lifetime risk of LBC from exposures to benzo[a]pyrene
LR_3_	Lifetime risk of LBC from occupational exposure
LR_4_	Lifetime risk of LBC from others risk factors
LR_o_	Lifetime risk of LBC from all risk factors for a 75-year-old (i.e., the cumulative incidence rate)
LRP_1_	Lifetime risk proportion from exposure to radon
LRP_2_	Lifetime risk proportion from exposure to benzo[a]pyrene
LRP_3_	Lifetime risk proportion from occupational exposure
LRP_4_	Lifetime risk proportion from exposure to other risk factors
NA	Non-industrial area
OF	Occupancy factor
PAF	Attributable fraction for the population
PCP	The average proportion of all cancers in employees with pneumoconiosis
PLC	The average proportion of LBC from all cancers in employees with pneumoconiosis
PM	Particulate matter
POP	The average population in monitored regions in years 1996–2016
RCF	Radon dose conversion factor
Rn	Radon
SIR	Age-standardized incidence rate
UCR	Unit cancer risk
US EPA	United States Environmental Protection Agency
VAR	Constant volume activity of radon in the observe period
WHO	World Health Organization
